# Beyond screen use duration: the role of evening TV-timing on sleep in preschool-aged children

**DOI:** 10.3389/frsle.2026.1783027

**Published:** 2026-04-01

**Authors:** Isabel M. Wilder, Rebecca M. C. Spencer, Tracy Riggins

**Affiliations:** 1 Neurocognitive Development Lab, Neuroscience and Cognitive Science (NACS) Program, University of Maryland, College Park, MD, United States; 2 Somneurolab, Department of Psychological and Brain Sciences, University of Massachusetts, Amherst, MA, United States; 3 Neurocognitive Development Lab, Department of Psychology, University of Maryland, College Park, MD, United States

**Keywords:** bedtime, family routines, preschool, screen use, sleep duration

## Abstract

Sleep is a critical component of early childhood health, yet many preschool-aged children fail to obtain sufficient overnight sleep. Screen use contributes to insufficient sleep. However, most research thus far has focused on total screen time rather than the timing of screen exposure, which may be especially important for evening arousal, displacement of bedtime routines, and circadian regulation. The present study examined whether the delay between evening TV use and bedtime (“TV-to-bed delay”) and children's average daily TV use were associated with 24-h sleep duration in preschoolers. Parents of 137 typically developing 3–5-year-old children (*M* age = 3.81 years, *SD* = 0.53; 52.6% female; 62.8% White; mid- to high-SES urban sample) provided reports of their child's typical TV-to-bed delay, average TV exposure, average nap duration, and 24-h sleep duration. Partial Spearman's rho correlations were used to examine associations between TV-use variables and 24-h sleep duration while controlling for child age, average daily TV use, and average nap duration. Longer TV-to-bed delays were significantly associated with longer 24-h sleep duration (ρ = 0.20, *p* = 0.02). In contrast, average daily TV use was not significantly associated with 24-h sleep duration after adjustment (ρ = −0.14, *p* = 0.11). These findings highlight the importance of considering timing as a meaningful dimension of media exposure and suggest that simple behavioral adjustments—such as creating a longer buffer between evening TV use and bedtime—may support healthier sleep in young children.

## 1 Introduction

Recent national data indicate that children ages eight and younger spend an average of 2 h and 27 min per day with screen media, with 2–4-year-olds averaging 2 h and 8 min daily (Rideout, 2025), exceeding the recommended screen time limit for this age group (1 h per day; World Health Organization, 2019). A 2020 report by Pew Research Center found that 74% of parents with children ages 2 years and under reported their child engaged with TV, with this number rising to 90% for children ages 3–4 years (Auxier et al., 2020). This increase has occurred alongside a proliferation of digital content aimed at young audiences, often under the assumption that it is educational. However, there are growing concerns about potential trade-offs for screen use, particularly in terms of its potential displacement of activities such as sleep, unstructured play, and caregiver interaction (American Academy of Pediatrics Council on Communications Media, 2016). Recent frameworks emphasize that sleep, sedentary behavior, and physical activity should be considered collectively within a 24-h movement paradigm, as these behaviors are interdependent and compete for time within a fixed day (World Health Organization, 2019). From this perspective, screen use may influence sleep not only through total duration but also through its placement within the daily behavioral sequence.

Sleep health is a multidimensional construct that is characterized by adequate sleep duration, sleep efficiency (i.e., sleep time relative to time in bed), appropriate timing, daytime alertness, and subjective satisfaction with overall sleep quality (Buysse, 2014). Based on substantial evidence that screen use impairs sleep in both children and adolescents, a recent consensus statement was issued on the impact of screen use on sleep across the lifespan (Hartstein et al., 2024). Recent studies have begun to explore relations between screen use and sleep in early childhood. For example, Hiltunen et al. (2021) showed that greater screen exposure in Finnish preschool-aged children (3–6 years, *n* = 736) was associated with later bedtimes and shorter sleep duration. Using a different national sample and objective sleep measurement, Kahn et al. (2021) demonstrated similar associations in Israeli preschoolers, showing that higher total screen exposure was linked to shorter sleep duration, later sleep onset, and poorer sleep efficiency in preschoolers assessed with actigraphy. Taken together, these studies highlight a consistent pattern: across multiple methodologies and populations, greater screen exposure in early childhood is linked to shorter and more disrupted sleep.

Although prior work consistently links greater screen exposure to poorer sleep in early childhood, most studies have operationalized screen use in terms of *overall daily duration* rather than the timing of when screen use ends relative to bedtime. For example, Hiltunen et al. (2021) and Kahn et al. (2021) quantified children's screen exposure as total daily use and demonstrated associations with shorter sleep duration and later sleep timing, but neither study examined the temporal proximity of screen use to bedtime. The distinction between screen quantity and screen timing is theoretically important, as models of sleep regulation suggest that screen exposure close to bedtime may exert unique effects on physiological arousal, bedtime routines, and melatonin secretion (Hale and Guan, 2015; Higuchi et al., 2005).

A smaller body of work has begun to examine screen exposure occurring closer to sleep onset. In early childhood, Helm and Spencer (2019) showed that the presence of a bedroom TV in 2.8–5.9-year-old children (*n* = 470) was associated with watching TV immediately before bed, later bedtimes, and approximately 30 min less nighttime sleep, suggesting that screen exposure occurring at the end of the evening routine may be particularly disruptive. Similarly, Staples et al. (2021) found that greater screen use during the hour before bedtime was associated with later sleep timing, shorter sleep duration, and greater night-to-night variability in 2.5-year-old children (*n* = 474). In older age groups, both observational (Hartley et al., 2022; Zhong et al., 2025) and experimental evidence (Perrault et al., 2019) further indicate that evening or after-bedtime screen use disrupts sleep timing, duration, and quality. However, even when screen exposure has been examined near bedtime, it has typically been operationalized using broad categories (e.g., screen use within a fixed pre-bedtime window or the presence of a bedroom TV), rather than as a direct measure of the *length of the delay* between screen cessation and bedtime. As a result, it remains unclear whether the closeness of screen use to bedtime contributes uniquely to sleep outcomes, independent of children's overall screen exposure.

Importantly, early childhood represents a period of substantial maturation in the systems underlying circadian rhythms, sleep pressure, and self-regulation. Between ages 3–5 years, many children transition away from daytime napping, which increases the importance of consolidated overnight sleep for cognitive and behavioral functioning (Riggins and Spencer, 2020). During this period, children also become more responsive to external cues such as routines, parental scaffolding (Mindell et al., 2015), and light exposure (Hartstein et al., 2022, 2023). As a result, environmental disruptions—such as late-evening screen use—may exert stronger effects on sleep than they do during later childhood.

Despite this heightened sensitivity, prior research has rarely differentiated *how much* screen use children receive from *when* that use occurs within the day. Many studies summarize exposure using total daily minutes, even though screen use at non-sleep-related times (e.g., morning cartoons or daytime educational content) may have very different implications than screen use occurring close to sleep onset. By directly measuring the delay between evening TV cessation and bedtime, the present study addresses this methodological gap and clarifies whether screen timing contributes uniquely to sleep duration beyond overall screen exposure.

From a translational perspective, identifying modifiable aspects of evening routines is essential for informing pediatric sleep recommendations. Reducing overall screen time can be challenging for families given the practical constraints of daily routines and the widespread presence of screens in modern households. In contrast, adjusting the timing of screen use within the evening routine may represent a more feasible and scalable intervention target. Understanding whether screen timing uniquely predicts sleep outcomes therefore has important implications for supporting healthy sleep in early childhood.

## 2 Methods

### 2.1 Participants

Data were drawn from two independent longitudinal studies examining sleep, memory, and brain development in early childhood. The first study, which has since concluded data collection (Allard et al., 2019; St. Laurent et al., 2022, 2025), and the second, an ongoing longitudinal study conducted across two collaborating sites (University of Maryland and the University of Massachusetts Amherst), assessed children at approximately 6-month intervals using comparable recruitment procedures and harmonized assessment protocols. For the present analyses, only wave 1 data from each study were included.

Participants from both studies were recruited from a large metropolitan area primarily through community advertisements and online postings. Interested caregivers contacted the research team and completed a screening procedure to determine eligibility.

Across both studies, children were required to be habitual nappers at enrollment (defined as ≥5 naps per week based on parent report). Children in the completed study were between 3 and 6 years of age at enrollment, whereas children in the ongoing study were between 3 and 4.5 years of age at enrollment.

Exclusion criteria across studies were designed to ensure a typically developing sample and included a history of head trauma; brain abnormality; neurological disorder; psychiatric disorder; developmental delay or diagnosed developmental disability; learning disability; neurodevelopmental disorder; sibling or parent diagnosis of autism; and premature birth (< 35 weeks gestation); diagnosed sleep disorder; current use of psychotropic or sleep-altering medications; recent travel outside the local time zone; acute illness at the time of testing; physical impairments interfering with assessment (e.g., vision or hearing not corrected to normal); and presence of metal in the body or other contraindications for MRI.

Across studies, 138 children had relevant data. After excluding one sleep-duration outlier (>3 SDs below the sample mean), the final analytic sample consisted of 137 preschool-aged children.

All procedures were reviewed and approved by the Universities' Institutional Review Boards. Informed consent was obtained from parents or legal guardians prior to participation, and all procedures were conducted in accordance with institutional guidelines.

### 2.2 Measures

#### 2.2.1 TV-to-bedtime delay (TV-to-bed delay)

Parents reported how long before bedtime their child typically stopped watching TV using a standardized categorical item. Response options ranged from: 1 = falls asleep with TV on; 2 = finishes TV immediately before bed; 3 = 30 min before bed; 4 = 1 h before bed; 5 = 2 h before bed; 6 = 3 h before bed, 7 = does not watch TV. Responses were treated as an ordinal measure reflecting increasing temporal distance between TV use and bedtime, rather than as a continuous time variable, because response options differed qualitatively (e.g., falling asleep with the TV on vs. stopping immediately before bed) and did not represent equal intervals.

#### 2.2.2 Average daily TV exposure

Parents reported their child's average daily TV exposure using two categorical items assessing time spent actively watching TV or videos on weekdays and weekends (response options: none, < 1 h, 1–3 h, 4–6 h, 7+ h per day). Responses were averaged across weekday and weekend items to create a composite measure of average daily TV exposure, which was treated as an ordinal variable. This measure reflects children's active TV viewing and is distinct from separate items assessing general household TV use (i.e., whether the TV is on regardless of child viewing). This parent-report format is widely used in early childhood research and provides a practical, developmentally appropriate estimate of children's typical daily screen exposure (Duch et al., 2013). Parent report is particularly appropriate for TV viewing in this age group, as televisions remain the most common device for video consumption in early childhood, with children spending more time watching videos on TVs than on mobile devices (Rideout, 2025). Much of this viewing occurs within shared household environments, including within the sleep environment (e.g., bedroom TVs; Helm and Spencer, 2019), providing caregivers substantial opportunity to observe viewing patterns.

#### 2.2.3 Overnight sleep duration

Parents reported their child's usual total amount of sleep each day (in hours), combining nighttime sleep and naps, as part of the Child Sleep Habits Questionnaire (Owens et al., 2000). This measure reflects parent-estimated sleep obtained rather than time spent in bed. Parents were instructed to consider their child's typical bedtime and wake time when estimating overnight sleep, ensuring that the measure reflected stable sleep patterns. This total reported was used as an index of 24-h sleep duration. Additionally, parents separately reported the typical duration of their child's nap (in hours), which was examined as a covariate in analyses.

### 2.3 Analyses

Analyses were conducted in R (Version 4.4.2; R Core Team, 2024) using RStudio (Version 2024.12.0; Posit Software, PBC, 2024). Descriptive statistics were computed for all study variables. Visual inspection of *Q*–*Q* plots indicated that continuous variables were approximately normally distributed, with only minor deviations at the distribution tails. However, because the primary predictors were ordinal, nonparametric Spearman correlations were used for primary analyses. Associations among TV-to-bed delay (ordinal), average daily TV use (ordinal), 24-h sleep duration (ratio), average nap duration (ratio), and age (ratio) were examined using partial Spearman's rho correlations. Partial correlations assessed the association between TV-to-bed delay and sleep duration while controlling for child age, average daily TV use, and average nap duration, as well as the association between average daily TV use and sleep duration while controlling for age, average nap duration, and TV-to-bed delay. All analyses were conducted using complete cases and evaluated using two-tailed significance tests, with statistical significance set at *p* < 0.05.

## 3 Results

Participants were preschool-aged children (ages 3–5 years) with roughly equal representation of females and males; detailed demographic characteristics are presented in Table 1.

**Table 1:** Demographic characteristics and descriptive statistics for participants (*n* = 137).

Demographic characteristic	%
Child sex	
Female	52.6
Male	47.4
Race (could select more than one)	
Asian	5.8
Black/African American	11.7
White or Caucasian	62.8
Did not disclose or other	2.9
Multiracial	16.7
Ethnicity	
Hispanic or Latino	19.1
16-7.4,-13.5242ptParental education (at least one parent with a 4-year college or higher)	89.7
Income	
< $25,000	5.8
$25,001–$55,000	11.1
$55,001–$105,000	36.8
>$105,000	46.2

M, mean; SD, standard deviation.

Descriptive statistics for all study variables are presented in Table 2. Zero-order Spearman correlations among study variables are presented in Table 2. Longer TV-to-bed delay ratings were significantly associated with longer 24-h sleep duration (ρ = 0.22, *p* < 0.05) and lower average daily TV use ratings (ρ = −0.17, *p* < 0.05). Average nap duration was positively associated with 24-h sleep duration (ρ = 0.33, *p* < 0.001) and negatively associated with child age (ρ = −0.23, *p* < 0.01). Greater average daily TV use ratings were not significantly associated with 24-h sleep duration (ρ = −0.15, *p* = 0.08). Age was not significantly associated with 24-h sleep duration (ρ = −0.07, *p* = 0.42), with average TV use ratings (ρ = 0.12, *p* = 0.15), or with the TV-to-bed delay ratings (ρ = −0.11, *p* = 0.21).

**Table 2:** Descriptive statistics for and correlations between study variables.

Variable	*M*	SD	Range	1	2	3	4	5	6
1. Age (years)	3.81	0.53	3.03–5.43	–					
2. Sex	–	–	–	−0.10	–				
3.24-h sleep duration	12.03	1.40	8.5–15.5	−0.07	0.08	–			
4. Avg nap duration (h)	1.66	0.52	0–3.5	−0.23^**^	0.26^**^	0.33^***^	–		
5. TV-to-bed delay (rating)	4.54	1.60	1–7	−0.11	0.05	0.22^*^	0.07	–	
6. Avg TV (rating)	2.49	0.66	1–4.5	0.12	0.12	−0.15	−0.05	−0.17^*^	–

Values represent Spearman correlation coefficients;

^*^p < 0.05,

^**^p < 0.01,

^***^p < 0.001.

TV-to-bed delay ratings ranged from 1 = falls asleep with TV on to 7 = does not watch TV, with higher values indicating a longer delay between TV cessation and bedtime. Average TV ratings ranged from 1 = none to 5 = 7+ h per day. Sex was coded 0 = female, 1 = male.

To evaluate the ordinal structure of the TV-to-bed delay variable, category means were examined. Children in the shortest-delay category obtained relatively less sleep (*M* = 11.75 h) whereas those in the longest delay category obtained the most sleep (*M* = 12.88 h), and sleep duration generally increased across longer-delay categories. Although the trend was not strictly linear across all categories, the association was not driven solely by the most extreme category. These findings are broadly consistent with a monotonic pattern, though the possibility of a threshold effect cannot be excluded.

**Figure 1:**
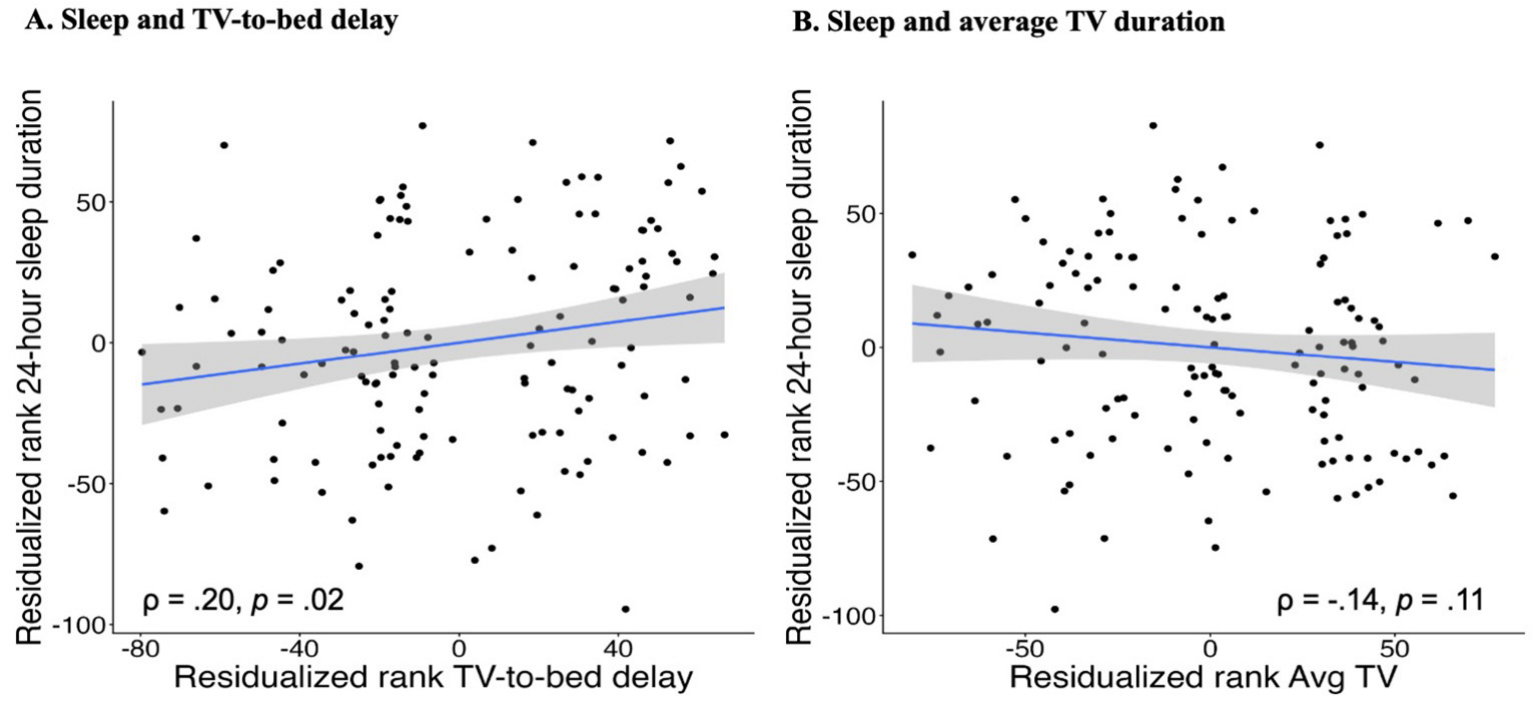
Partial regression plots predicting sleep duration from TV use. **(A)** Sleep and TV-to-bed delay. **(B)** Sleep and average TV duration. Scatterplots depict partial Spearman associations between screen-use variables and 24-h sleep duration, visualized using rank-transformed variables with covariate effects removed. Panel **(A)** shows the significant positive association between residualized rank TV-to-bed delay and residualized rank 24-h sleep duration after adjusting for child age, average daily TV use, and average nap duration. Panel **(B)** shows the non-significant association between residualized rank average daily TV use and residualized rank 24-h sleep duration after adjusting for child age, TV-to-bed delay, and average nap duration. Shaded regions represent 95% confidence intervals.

To examine whether the association between TV-to-bed delay ratings and 24-h sleep duration was independent of child age, average TV exposure ratings, and average nap duration, partial Spearman's rho correlations were conducted. Longer TV-to-bed delay ratings were significantly associated with longer overnight sleep duration after controlling for age, average daily TV use ratings, and average nap duration (ρ = 0.20, *p* = 0.02; Figure 1A). In contrast, average daily TV use ratings were not significantly associated with 24-h sleep duration after controlling for age, TV-to-bed delay ratings, and average nap duration (ρ = −0.14, *p* = 0.11; Figure 1B).

To evaluate potential sex differences, a TV-to-bed delay by sex interaction was tested in a regression model predicting 24-h sleep while controlling for age, average TV use, and average nap duration. Neither sex nor the interaction was significant (*ps* > 0.50), indicating that the association between TV-to-bed delay and sleep duration did not differ by sex.

## 4 Discussion

The present study examined whether the timing of TV use is associated with sleep duration in preschool-aged children. Consistent with prior work linking screen exposure to poorer sleep outcomes in early childhood (Hiltunen et al., 2021), the current findings indicate that the time at which TV use ends relative to sleep onset may be more important for sleep duration than how much TV children watch overall. Specifically, longer delays between ending TV use and bedtime were associated with longer 24-h sleep duration, even after accounting for child age and average daily TV exposure. In contrast, total daily TV use was not independently associated with sleep duration once timing was considered. Although the observed associations were statistically significant, the effect sizes were small in magnitude (partial ρ = 0.20); this corresponds to approximately 4% of the variance in 24-h sleep duration, indicating that TV-to-bed delay accounts for only a small proportion of variance in sleep duration. Thus, although the relation is detectable at the population level, it may not translate into large differences in sleep for every individual child.

These findings are most closely aligned with prior studies examining screen exposure in close temporal proximity to bedtime. For example, Helm and Spencer (2019) reported that preschool-aged children with televisions in their bedrooms—particularly those who fell asleep with the TV on—obtained less sleep and experienced later sleep timing. Similarly, Staples et al. (2021) showed that screen use during the hour before bedtime was associated with later sleep onset and greater night-to-night variability. The current results are consistent with these studies and suggest that screen use occurring close to bedtime may be especially disruptive for sleep. By isolating the duration of the buffer between TV cessation and bedtime, the present findings extend this work by suggesting that even modest increases in pre-bedtime screen-free time may be associated with longer sleep duration.

One important consideration is that the observed association between TV-to-bed delay and sleep duration may be influenced in part by children with very short delays between TV cessation and bedtime. Because TV-to-bed delay was measured categorically, it is not possible to determine whether the association reflects a graded dose-response relationship or a threshold effect in which screen exposure occurring very close to bedtime is particularly disruptive. Scatterplots suggest that associations may be influenced by shorter delays, underscoring the need for future studies using finer-grained or continuous measures of screen timing to more precisely characterize this association.

More broadly, the present results build on a substantial body of research demonstrating associations between overall screen exposure and sleep outcomes in preschool-aged children. Prior studies have shown that greater total screen time is associated with shorter sleep duration and later bedtimes across diverse samples and methodologies (e.g., Kahn et al., 2021; Hiltunen et al., 2021). However, much of this work has operationalized screen exposure primarily in terms of daily duration, making it difficult to disentangle the effects of quantity from those of timing. The current findings suggest that associations between total screen time and sleep may, in part, reflect when screens are used in the evening, rather than cumulative exposure alone.

The observed association between television timing and overnight sleep duration is consistent with theoretical models emphasizing the particular sensitivity of sleep-related processes to late-day screen exposure. Evening screen use may delay sleep by increasing cognitive or emotional arousal, displacing bedtime routines, or interfering with circadian regulation through exposure to light (Hale and Guan, 2015; Higuchi et al., 2005). Experimental work in preschool-aged children (3–5 years) has demonstrated that light exposure during the hour before habitual bedtime can suppress melatonin secretion by 70%−99%, suggesting heightened light sensitivity during early childhood (Hartstein et al., 2022). Although the present study did not directly assess physiological mechanisms, the behavioral findings align with this experimental evidence by showing that children who stopped watching television earlier tended to obtain longer reported sleep, even when total screen exposure was held constant. At the same time, the relatively small effect sizes suggest that screen timing represents only one of many factors influencing sleep duration in preschool-aged children. Other unmeasured influences—including broader bedtime routines, parenting practices, household structure, and environmental factors—likely play substantial roles.

From a practical perspective, distinguishing between screen timing and screen duration may have important implications for families and clinicians. Reducing overall screen time can be challenging to implement, particularly in households where TV use is embedded in daily routines. In contrast, encouraging a longer buffer between TV use and bedtime may represent a promising target for future intervention studies, although the present findings do not allow for causal conclusions to be drawn. Together, these findings highlight screen timing as a potentially important dimension of media exposure in early childhood that requires further longitudinal and experimental investigation.

The results also align with broader developmental research showing that preschoolers rely heavily on environmental cues to regulate sleep, with consistent bedtime routines supporting physiological wind-down processes (Mindell et al., 2015). Importantly, however, the presence of a routine alone does not guarantee regulatory benefit. When screen use is incorporated into the bedtime routine itself, the routine may undermine the regulatory function it is intended to serve by introducing competing sources of cognitive and emotional arousal. Screens may disrupt bedtime sequencing not only through light exposure but also through heightened cognitive and emotional engagement that can prolong arousal and delay sleep readiness. For example, fast-paced or emotionally stimulating content may make it more difficult for children to transition into a restful state, even when screen use is predictable or habitual. From this perspective, measuring the TV-to-bed delay provides a more targeted indicator of whether bedtime routines include a screen-free buffer that supports wind-down, rather than simply whether a routine exists.

Despite these strengths, several limitations must be considered. First, the reliance on parent-reported measures introduces the possibility of recall bias, particularly for screen timing. Although parent-report measures of TV-to-bed delay are widely used, more precise time-stamped assessments (e.g., digital logs) would improve measurement precision, as parents may not be accurate reporters of children's television viewing (Wood et al., 2019). Second, the sample was predominantly White and highly educated, which may limit generalizability. Families with fewer socioeconomic resources may have different evening routines or different reliance on screens for household management. Third, the cross-sectional design prevents causal inference. It is possible, for example, that children who naturally sleep longer have more structured routines that include earlier ending of screen use, rather than screen timing directly influencing sleep. Longitudinal data or randomized interventions manipulating screen timing would help clarify directionality. Fourth, although the sample size was adequate for detecting small-to-moderate correlations, it may have limited statistical power to detect smaller effects, particularly for associations involving average daily TV use. The non-significant findings for average TV use should therefore be interpreted cautiously, as small associations may not have been detectable in the present sample. Larger samples would allow for more precise effect size estimation. Finally, the present study did not directly assess markers of homeostatic sleep pressure or sleep timing (e.g., bedtime clock time, sleep onset latency, variability across nights). Particularly during the preschool years—when children are transitioning out of regular napping—homeostatic regulation of sleep pressure undergoes developmental change (Kurth et al., 2016). Without measures of sleep timing or pressure, it remains unclear whether the observed association reflects effects on sleep duration, shifts in sleep phase, or broader regulation of sleep pressure across the day.

Future research should incorporate objective measures of sleep (e.g., actigraphy) alongside day-by-day assessments of screen use (e.g., daily diaries or ecological momentary assessment), which would allow investigators to disentangle acute effects of screen exposure occurring close to bedtime from more chronic or cumulative patterns of use. Examining the interaction between screen content (educational vs. fast-paced), context (co-viewing vs. solo), and timing may also yield a more nuanced understanding of media effects. Additionally, testing these associations in more diverse samples, including families from a broader socioeconomic range, will be important for assessing generalizability and equity of sleep recommendations.

Overall, the present study contributes to a growing body of evidence suggesting that the timing of evening screen use is associated with sleep duration in early childhood. By drawing attention to media timing as a distinct aspect of media exposure, this research identifies an important area for continued work examining whether modifications to evening routines are linked to changes in sleep outcomes and broader developmental trajectories in childhood.

## Data Availability

The raw data supporting the conclusions of this article will be made available by the authors, without undue reservation.

## References

[B1] Allard T. Riggins T. Ewell A. Weinberg B. Lokhandwala S. Spencer R. M. C. (2019). Measuring neural mechanisms underlying sleep-dependent memory consolidation during naps in early childhood. J. Vis. Exp. 152:e60200. doi: 10.3791/60200 31633692 PMC8340910

[B2] American Academy of Pediatrics Council on Communications and Media (2016). Media and young minds. Pediatrics 138:e20162591. doi: 10.1542/peds.2016-2591 27940793

[B3] Auxier B. Anderson M. Perrin A. Turner E. (2020). Children's Engagement with Digital Devices, Screen Time. Pew Research Center. Available online at: https://www.pewresearch.org/internet/2020/07/28/childrens-engagement-with-digital-devices-screen-time/ (Accessed November 10, 2025).

[B4] Buysse D. J. (2014). Sleep health: can we define it? Does it matter? Sleep 37, 9–17. doi: 10.5665/sleep.3298 24470692 PMC3902880

[B5] Duch H. Fisher E. M. Ensari I. Harrington A. (2013). Screen time use in children under 3 years old: a systematic review of correlates. Int. J. Behav. Nutr. Phys. Act. 10:102. doi: 10.1186/1479-5868-10-102 23967799 PMC3844496

[B6] Hale L. Guan S. (2015). Screen time and sleep among school-aged children and adolescents: a systematic literature review. Sleep Med. Rev. 21, 50–58. doi: 10.1016/j.smrv.2014.07.007 25193149 PMC4437561

[B7] Hartley S. Royant-Parola S. Zayoud A. Gremy I. Matulonga B. (2022). Do both timing and duration of screen use affect sleep patterns in adolescents? PLoS One 17:e0276226. doi: 10.1371/journal.pone.0276226 36264928 PMC9584513

[B8] Hartstein L. E. Behn C. D. Akacem L. D. Stack N. Wright K. P. LeBourgeois M. K. (2022). High sensitivity of melatonin suppression response to evening light in preschool-aged children. J. Pineal Res. 72:e12780. doi: 10.1111/jpi.12780 34997782 PMC8933063

[B9] Hartstein L. E. Diniz Behn C. Wright K. P. Jr. Akacem L. D. Stowe S. R. LeBourgeois M. K. (2023). Evening light intensity and phase delay of the circadian clock in early childhood. J. Biol. Rhythms 38, 77–86. doi: 10.1177/07487304221134330 36415902 PMC11302507

[B10] Hartstein L. E. Mathew G. M. Reichenberger D. A. Rodriguez I. Allen N. Chang A.-M. . (2024). The impact of screen use on sleep health across the lifespan: a National Sleep Foundation consensus statement. Sleep Health 10, 373–384. doi: 10.1016/j.sleh.2024.05.001 38806392 PMC13181348

[B11] Helm A. F. Spencer R. M. C. (2019). Television use and its effects on sleep in early childhood. Sleep Health 5, 241–247. doi: 10.1016/j.sleh.2019.02.009 30987948 PMC6581597

[B12] Higuchi S. Motohashi Y. Liu Y. Maeda A. (2005). Effects of playing a computer game using a bright display on presleep physiological variables, sleep latency, slow wave sleep and REM sleep. J. Sleep Res. 14, 267–273. doi: 10.1111/j.1365-2869.2005.00463.x 16120101

[B13] Hiltunen P. Leppänen M. H. Ray C. Määttä S. Vepsäläinen H. Koivusilta L. . (2021). Relationship between screen time and sleep among Finnish preschool children: results from the DAGIS study. Sleep Med. 77, 75–81. doi: 10.1016/j.sleep.2020.11.008 33338700

[B14] Kahn M. Schnabel O. Gradisar M. Rozen G. S. Slone M. Atzaba-Poria N. . (2021). Sleep, screen time and behaviour problems in preschool children: an actigraphy study. Eur. Child Adolesc. Psychiatry 30, 1793–1802. doi: 10.1007/s00787-020-01654-w 33006004

[B15] Kurth S. Lassonde J. M. Pierpoint L. A. Rusterholz T. Jenni O. G. McClain I. J. . (2016). Development of nap neurophysiology: preliminary insights into sleep regulation in early childhood. J. Sleep Res. 25, 646–654. doi: 10.1111/jsr.12427 27252144 PMC5135687

[B16] Mindell J. A. Li A. M. Sadeh A. Kwon R. Goh D. Y. T. (2015). Bedtime routines for young children: a dose-dependent association with sleep outcomes. Sleep 38, 717–722. doi: 10.5665/sleep.4662 25325483 PMC4402657

[B17] Owens J. A. Spirito A. McGuinn M. (2000). The children's sleep habits questionnaire (CSHQ): psychometric properties of a survey instrument for school-aged children. Sleep 23, 1043–1051. doi: 10.1093/sleep/23.8.1d 11145319

[B18] Perrault A. A. Bayer L. Peuvrier M. Afyouni A. Ghisletta P. Brockmann C. . (2019). Reducing the use of screen electronic devices in the evening is associated with improved sleep and daytime vigilance in adolescents. Sleep 42:zsz125. doi: 10.1093/sleep/zsz125 31260534

[B19] Posit Software PBC. (2024). RStudio: Integrated Development Environment for R. Boston, MA: Posit Software, PBC. Available online at: https://posit.co/ (Accessed November 10, 2025).

[B20] R Core Team (2024). R: A Language and Environment for Statistical Computing. R Foundation for Statistical Computing. Available online at: https://www.R-project.org/ (Accessed November 10, 2025).

[B21] Rideout V. (2025). The Common Sense Census: Media Use by Kids Zero to Eight, 2025. Common Sense Media. Available online at: https://www.commonsensemedia.org/sites/default/files/research/report/2025-common-sense-census-web-2.pdf (Accessed March 1, 2026).

[B22] Riggins T. Spencer R. M. C. (2020). Habitual sleep is associated with both source memory and hippocampal subfield volume during early childhood. Sci. Rep. 10:15304. doi: 10.1038/s41598-020-72231-z 32943722 PMC7499159

[B23] Staples A. D. Hoyniak C. McQuillan M. E. Molfese V. Bates J. E. (2021). Screen use before bedtime: consequences for nighttime sleep in young children. Infant Behav. Dev. 62:101522. doi: 10.1016/j.infbeh.2020.101522 33385752 PMC7977486

[B24] St. Laurent C. W. Lokhandwala S. Allard T. Ji A. Paluch A. Riggins T. . (2025). Relations between 24-h movement behaviors, declarative memory, and hippocampal volume in early childhood. Sci. Rep. 15:9205. doi: 10.1038/s41598-025-92932-7 40097472 PMC11914694

[B25] St. Laurent C. W. Lokhandwala S. Allard T. Ji A. Riggins T. Spencer R. M. C. (2022). Influence of naps on sedentary time and physical activity in early childhood. Sci. Rep. 12:21198. doi: 10.1038/s41598-022-25628-x 36482180 PMC9731956

[B26] Wood C. T. Skinner A. C. Brown J. D. Brown C. L. Howard J. B. Steiner M. J. . (2019). Concordance of child and parent reports of children's screen media use. Acad. Pediatr. 19, 529–533. doi: 10.1016/j.acap.2019.04.002 30981024 PMC6612580

[B27] World Health Organization (2019). Guidelines on Physical Activity, Sedentary Behaviour and Sleep for Children under 5 Years of Age. Available online at: https://www.who.int/publications/i/item/9789241550536 (Accessed November 10, 2025). 31091057

[B28] Zhong C. Masters M. Donzella S. M. Diver W. R. Patel A. V. (2025). Electronic screen use and sleep duration and timing in adults. JAMA Netw Open 8:e252493. doi: 10.1001/jamanetworkopen.2025.2493 40146105 PMC11950897

